# Suicidal Ideation and Attempts Among Students in Grades 8, 10, and 12 — Utah, 2015

**DOI:** 10.15585/mmwr.mm6715a4

**Published:** 2018-04-20

**Authors:** Marissa L. Zwald, Francis B. Annor, Amanda Wilkinson, Mike Friedrichs, Anna Fondario, Angela C. Dunn, Allyn Nakashima, Leah K. Gilbert, Asha Ivey-Stephenson

**Affiliations:** ^1^Epidemic Intelligence Service, CDC; ^2^Division of Health and Nutrition Examination Surveys, National Center for Health Statistics, CDC; ^3^Division of Violence Prevention, National Center for Injury Prevention and Control, CDC; ^4^Child Health and Mortality Prevention Surveillance (CHAMPS), Center for Global Health, CDC; ^5^Utah Department of Health, Salt Lake City, Utah.

Suicidal thoughts and behaviors among youths are important public health concerns in Utah, where the suicide rate among youths consistently exceeds the national rate and has been increasing for nearly a decade (*1*). In March 2017, CDC was invited to assist the Utah Department of Health (UDOH) with an investigation to characterize the epidemiology of fatal and nonfatal suicidal behaviors and identify risk and protective factors associated with these behaviors, among youths aged 10–17 years. This report presents findings related to nonfatal suicidal behaviors among Utah youths. To examine the prevalence of suicidal ideation and attempts among Utah youths and evaluate risk and protective factors, data from the 2015 Utah Prevention Needs Assessment survey were analyzed. Among 27,329 respondents in grades 8, 10, and 12, 19.6% reported suicidal ideation and 8.2% reported suicide attempts in the preceding 12 months. Significant risk factors for suicidal ideation and attempts included being bullied, illegal substance or tobacco use in the previous month, and psychological distress. A significant protective factor for suicidal ideation and attempts was a supportive family environment. UDOH, local health departments, and other stakeholders are using these findings to develop tailored suicide prevention strategies that address multiple risk and protective factors for suicidal ideation and attempts. Resources such as CDC’s *Preventing Suicide: A Technical Package of Policy, Programs, and Practices* (*2*) can help states and communities identify strategies and approaches using the best available evidence to prevent suicide, which include tailored strategies for youths.

The Utah Prevention Needs Assessment is a cross-sectional, school-based health and risk behavior survey conducted biennially in randomly selected public and charter schools in Utah among a representative sample of students in grades 6, 8, 10, and 12 (*3*). The survey is anonymous, and students are required to have parental consent to participate. The school sample is stratified by district; data were weighted to account for the probability of selection and the distribution of students by sex, grade, and race using iterative proportional fitting. Additional survey details are available elsewhere (*3*). Among 75,652 youths sampled for the 2015 Utah Prevention Needs Assessment survey, 48,975 (64.7%) participated. For this analysis, 29,089 students aged <18 years in grades 8, 10, and 12 were considered eligible. Approximately 6% of eligible participants were excluded because of missing outcome data, yielding a final analytic sample of 27,329.

Suicidal ideation was defined as an affirmative response to either of the following questions: “During the past 12 months, did you ever seriously consider attempting suicide?” (Yes or No) or “During the past 12 months, did you make a plan about how you would attempt suicide?” (Yes or No). Suicidal attempt was assessed by the response to the question “During the past 12 months, how many times did you actually attempt suicide?” Response options were 0, 1, 2–3, 4–5, or ≥6 times. Because of a skewed distribution, where a small percentage of youths reported multiple suicide attempts (4.2% reported 1; 2.6% reported 2–3; 0.7% reported 4–5; and 0.7% reported ≥6), responses were dichotomized to none (zero times) and ≥1 (≥1 time). Data from additional questions were used to measure risk factors, including bullying on school property in the previous year, electronic bullying in the previous year, any illicit substance use in the previous month, any tobacco use in the previous month, and psychological distress. Protective factors assessed were perceptions of prosocial behaviors and separate measures for a supportive community, school, peer, and family environment (*4*). Data were analyzed by selected demographic characteristics and weighted to provide estimates of suicidal ideation and attempts with accompanying 95% confidence intervals (CIs). Multivariate logistic regression analyses were conducted to examine risk and protective factors associated with suicidal ideation and attempts in the previous 12 months controlling for all other factors and demographic characteristics informed by prior research (*5*–*10*): sex, age, race, religious preference, and highest level of education in the household. Adjusted odds ratios (AORs) and 95% CIs were calculated, with p<0.05 considered statistically significant. Variables in the final models were screened for multicollinearity. Statistical software was used to account for the complex survey design.

In 2015, almost 20% of students in grades 8, 10, and 12 who participated in the Utah Prevention Needs Assessment survey reported suicidal ideation and 8.2% reported having attempted suicide during the past 12 months (Table 1). Prevalence of suicidal ideation and attempts were highest among students who were female, aged 15–17 years, in grade 10, nonwhite, less religious, nonmembers of the Church of Latter Day Saints, and had a household education attainment level of less than high school. After adjusting for the other factors and for demographic characteristics, odds of suicidal ideation were higher among students who were bullied on school property (AOR = 1.95; 95% CI = 1.54–2.48) or electronically (1.82; 1.46–2.26) in the previous year, who reported illicit substance use (1.93; 1.42–2.62) or tobacco use (1.54; 1.14–2.09) in the previous month, and who reported moderate psychological distress (5.67; 4.42–7.28) or serious psychological distress (16.37; 12.12–22.10) (Table 2). Risk for suicide attempt was higher among students who were bullied on school property (2.17; 1.59–2.96), electronically bullied (1.71; 1.19–2.45), used an illicit substance in the previous month (1.90; 1.32–2.74), used tobacco in the previous month (1.70; 1.10–2.63), and reported moderate (3.80; 2.40–6.01) or serious (8.91; 5.75–13.80) psychological distress. A supportive family environment was protective against suicidal ideation (0.86; 0.83–0.90) and suicide attempts (0.87; 0.83–0.93). Nonsignificant protective factors for both suicidal ideation and suicide attempts included prosocial behaviors, and supportive community, school, and peer environments.

**TABLE 1:** Prevalence of suicidal ideation and attempt during the past 12 months among students in grades 8, 10, and 12, by selected characteristics — Utah Prevention Needs Assessment Survey, Utah, 2015

Characteristic	No. in sample (weighted %)	Suicidal ideation*			≥1 suicide attempt^†^		
		No.	% (95% CI)	p-value^§^	No.	% (95% CI)	p-value^§^
**Total**	**27,329**	**5,347**	**19.6 (18.6–20.7)**	**—**	**2,338**	**8.2 (7.6–8.8)**	**—**
**Sex**							
Male	12,706 (49.9)	1,680	13.7 (12.6–14.9)	<0.001	634	5.0 (4.4–5.6)	<0.001
Female	14,507 (50.1)	3,631	25.5 (24.1–27.0)		1,693	11.4 (10.5–12.4)	
**Age group (yrs)**							
12–14	13,111 (40.9)	2,399	17.0 (15.9–18.2)	<0.001	1,135	7.9 (7.1–8.7)	0.395
15–17	14,218 (59.1)	2,948	21.4 (20.0–22.9)		1,203	8.4 (7.6–9.3)	
**Grade**							
8	13,206 (41.2)	2,421	17.1 (15.9–18.3)	<0.001	1,148	7.9 (7.1–8.8)	0.001
10	10,616 (41.8)	2,299	22.6 (20.8–24.6)		977	9.4 (8.3–10.7)	
12	3,507 (17.1)	627	18.5 (16.7–20.6)		213	5.8 (4.8–7.1)	
**Race**							
White	21,988 (80.9)	4,095	18.7 (17.6–19.9)	<0.001	1,653	7.2 (6.6–7.9)	<0.001
Nonwhite	5,208 (19.1)	1,225	23.4 (21.7–25.2)		673	12.3 (11.2–13.4)	
**Religious attendance^¶^**							
Religious	17,479 (67.7)	2,762	16.1 (15.2–17.1)	<0.001	1,079	5.9 (5.3–6.5)	<0.001
Less religious	8,792 (32.2)	2,400	27.4 (25.9–29.1)		1,158	13.0 (11.9–14.2)	
Religious preference**							
LDS (Mormon)	16,120 (62.7)	2,398	15.3 (14.4–16.4)	<0.001	865	5.1 (4.6–5.7)	<0.001
Other	9,982 (37.3)	2,717	27.1 (25.5–28.7)		1,339	13.0 (11.9–14.2)	
**Highest household education level**							
Less than high school	1,561 (6.4)	472	27.6 (24.4–31.1)	<0.001	277	15.6 (13.2–18.3)	<0.001
High school graduate or some college	7,707 (30.8)	1,853	23.4 (21.9–25.0)		849	10.3 (9.2–11.4)	
College graduate	14,649 (62.7)	2,336	16.9 (15.8–18.1)		850	6.0 (5.4–6.7)	

**Abbreviations:** CI = confidence interval; LDS = Latter Day Saints.

* Suicidal ideation defined as a response of “yes” to either of the following questions: “During the past 12 months, did you ever seriously consider attempting suicide?” and “During the past 12 months, did you make a plan about how you would attempt suicide?”

^†^ Suicidal attempt was based on a question asking “During the past 12 months, how many times did you actually attempt suicide? Response options were 0 times, 1 time, 2–3 times, 4–5 times, or ≥6 times. Responses were dichotomized to none (0 times) and ≥1 (1 or more times).

^§^ p-value for Chi-square test.

^¶^ Based on a question asking “How often do you attend religious service or activities?” Responses of attends 1–2 times per month and about once a week or more were categorized as “religious” and responses of never and rarely were categorized as “less religious.”

** Based on a question asking “Which is your religious preference (choose the ONE religion with which you identify the most)?” Responses of Catholic, Jewish, Protestant, another religion, or no religious preference were categorized as “Other.”

**TABLE 2:** Adjusted odds ratios of suicidal ideation and suicide attempt during the preceding 12 months among students in grades 8, 10, and 12 — Utah Prevention Needs Assessment Survey, Utah, 2015

Characteristic	AOR* (95% CI)	
	Suicidal ideation^†^	Suicide attempt^§^
**Risk factor**		
Bullied on school property in the previous year^¶^	1.95 (1.54–2.48)	2.17 (1.59–2.96)
Electronically bullied in the previous year^¶^	1.82 (1.46–2.26)	1.71 (1.19–2.45)
Any substance use in the previous month**	1.93 (1.42–2.62)	1.90 (1.32–2.74)
Any tobacco use in the previous month**	1.54 (1.14–2.09)	1.70 (1.10–2.63)
**Psychological distress^††^**		
No distress	Referent	Referent
Moderate distress	5.67 (4.42–7.28)	3.80 (2.40–6.01)
Serious distress	16.37 (12.12–22.10)	8.91 (5.75–13.80)
**Protective factor**		
Prosocial behaviors^§§^	1.01 (0.99–1.03)	0.99 (0.96–1.02)
Supportive community environment^¶¶^	0.98 (0.94–1.02)	0.95 (0.90–1.01)
Supportive school environment***	1.03 (0.99–1.08)	1.05 (0.99–1.12)
Supportive peer environment^†††^	1.01 (1.00–1.03)	1.00 (0.97–1.02)
Supportive family environment^§§§^	0.86 (0.83–0.90)	0.87 (0.83–0.93)

**Abbreviations:** AOR = adjusted odds ratio; CI = confidence interval.

* Multivariate models adjusted for all other factors in the model and sex, age, race, religious preference, and highest education level of entire household.

^†^ Suicidal ideation defined as a response of “yes” to either of the following questions: “During the past 12 months, did you ever seriously consider attempting suicide?” and “During the past 12 months, did you make a plan about how you would attempt suicide?”

^§^ Suicide attempt was based on a question asking “During the past 12 months, how many times did you actually attempt suicide? Response options were 0 times, 1 time, 2–3 times, 4–5 times, or ≥6 times. Responses were dichotomized to none (0 times) and ≥1 (1 or more times).

^¶^ Referent = not bullied.

** Referent = no use. Substance use defined as using any alcohol, marijuana, or illicit drugs in the previous month. Tobacco use defined as using any tobacco product, including e-cigarettes, in the previous month.

^††^ Psychological distress was estimated using the Kessler K6 scale (https://www.ncbi.nlm.nih.gov/pubmed/12214795) which screens for psychological distress by asking students “During the past 30 days, how often did you (a) feel nervous, (b) feel hopeless, (c) feel restless or fidgety, (d) feel so depressed that nothing could cheer you up, (e) feel that everything was an effort, (f) feel worthless? Answers to each were scored based on the following responses: “None of the time” (0 points); “A little of the time” (1 point); “Some of the time” (2 points); “Most of the time” (3 points); and “All of the time” (4 points). The psychological distress variable was created by generating a composite score from the six items above. Students with a total score of ≥13 points were determined to have high psychological distress; students with a score of 7–12 points were considered to have moderate psychological distress; and students with a score of 0–6 points were considered to have no psychological distress (α = 0.91).

^§§^ For prosocial behaviors, a mean score was calculated from the following three items, which were on an 8-point Likert scale. Items asked included how many times in the past year “Have you participated in clubs, organizations, or activities at school?”; “Have you done extra work on your own for school?”; and “Have you volunteered to do community service?” A higher mean score indicated stronger prosocial behaviors, with a possible range of 1–24 (α = 0.70).

^¶¶^ For supportive community level environment, a mean score was calculated from the following three items, which were on a 4-point Likert scale. Statements included the following: “My neighbors notice when I am doing a good job and let me know about it”; “There are people in my neighborhood who are proud of me when I do something well”; and “There are people in my neighborhood who encourage me to do my best.” A higher mean score indicated a more supportive community level environment, with a possible range of 1–12 (α = 0.90).

*** For supportive school environment, a mean score was calculated from the following five items, which were on a 4-point Likert scale. Statements included the following: “In my school, students have lots of chances to help decide things like class activities and rules”; “There are lots of chances for students in my school to talk with a teacher one-on-one”; “My teachers notice when I am doing a good job and let me know about it”; “I have lots of chances to be part of class discussions or activities”; and “Teachers ask me to work on special classroom projects.” A higher mean score indicates a more supportive school environment, with a possible range of 1–20 (α = 0.69).

^†††^ For supportive peer environment, a mean score was calculated from five items, which were on a 5-point Likert scale. Items asked in the past year included how many of your best friends have “Participated in school clubs”; “Made a commitment to stay drug-free”; “Tried to do well in school”; “Have liked school”; and “Regularly attended religious services.” A higher mean score indicated a more supportive peer environment, with a possible range of 1–25 (α = 0.79).

^§§§^ For supportive family environment, a mean score was calculated from the following three items, which were on a 4-point Likert scale. Statements included the following: “My parents ask me what I think before most family decisions affecting me are made”; “If I had a personal problem, I could ask my mom or dad for help”; and “My parents give me lots of chances to do fun things with them.” A higher mean score indicated stronger family environments, with a possible range of 1–12 (α = 0.84).

## Discussion

Data from Utah’s largest school health and risk behavior survey on suicidal ideation and suicide attempts among students in grades 8, 10, and 12 indicate that in 2015, approximately one in five Utah youths reported suicidal ideation and 8.2% attempted suicide during the previous 12 months. Consistent with previous evidence and an investigation of youth suicide in California (*5*,*6*), nonfatal suicidal behaviors examined in the current investigation differed from those of completed suicides among Utah youths described elsewhere (*6*). For example, the prevalence of suicidal ideation and suicide attempts were highest among females and nonwhites, whereas rates of completed suicide among youths in Utah during 2011–2015 were higher among males (11.8 per 100,000 [95% CI = 9.7–14.0]) than among females (3.7 [2.5–5.1]) and among whites (8.3 [6.8–9.7]) than among nonwhites (6.5 [4.1–8.9]) (*7*). Past research has demonstrated similar sex differences in nonfatal and fatal suicidal behaviors among youths. Rates of suicidal ideation and suicide attempts are higher among adolescent females in the United States, whereas rates of completed suicide are higher among adolescent males, which is in part a consequence of the choice of more lethal suicide attempt methods among males (*1*,*8*–*10*).

Several factors were associated with a higher risk for suicidal thoughts and behaviors, including being bullied at school and online, recently using illicit substances and tobacco, and experiencing psychological distress. Youths with a supportive family environment had a lower risk for suicidal ideation and suicide attempts, which has been demonstrated in previous studies related to the family environment and suicidal thoughts and behaviors, where family cohesion, positive parent-child connection, time spent together, parental supervision, and high parental expectations of academics and behaviors were protective against suicidal behaviors (*8*,*9*). Public health professionals in Utah who are developing and implementing youth suicide prevention interventions might consider extending initiatives to the home environment to include family members and addressing protective and risk factors identified in this investigation.

The findings in this report are subject to at least three limitations. First, because the survey is cross-sectional in nature, whether the risk and protective factors assessed were precursors or consequences of suicidal ideation and attempts could not be determined. Second, these data apply only to students in grades 8, 10, and 12 who were attending Utah public and charter schools and are not representative of all persons in these grades. Finally, data are self-reported and possibly subject to underreporting or overreporting of suicidal thoughts and behaviors because of, for example, unwillingness to disclose certain experiences and recall bias.

Continued surveillance for suicidal thoughts and behaviors among Utah youths is important to planning, implementing, and evaluating public health interventions aimed at preventing youth suicide. Possible prevention strategies to consider could include integrating family members and the home setting into existing or new interventions and identifying and addressing the needs of youths exhibiting risk factors identified in this investigation (e.g., being bullied, using illegal substances or tobacco, or experiencing psychological distress). Resources such as CDC’s *Preventing Suicide: A Technical Package of Policy, Programs, and Practices* (*2*) can help states and communities identify strategies and approaches using the best available evidence to prevent suicide, which include tailored strategies for youths. Public health professionals and other stakeholders might consider employing the outlined strategies in the technical package to help address suicidal thoughts and behaviors among Utah youths.

**Summary**

**What is already known about this topic?**

The youth suicide rate in Utah is consistently higher than the national rate and has been increasing for nearly a decade.

**What is added by this report?**

In 2015, approximately 20% of youths in Utah considered suicide, and 8% attempted suicide. Youths who were bullied, reported recent illicit substance or tobacco use, and experienced psychological distress had a higher risk for suicidal ideation and attempts. Youths with a supportive family environment had a lower risk for suicidal thoughts and behaviors.

**What are the implications for public health practice?**

These results can help guide suicide prevention strategies in Utah and elsewhere. CDC’s evidence-based *Preventing Suicide: A Technical Package of Policy, Programs, and Practices *includes tailored strategies for youths.
